# Cancer Stem Cells and Their Interaction with the Tumor Microenvironment in Neuroblastoma

**DOI:** 10.3390/cancers8010005

**Published:** 2015-12-31

**Authors:** Evan F. Garner, Elizabeth A. Beierle

**Affiliations:** Department of Surgery, Division of Pediatric Surgery, University of Alabama, Birmingham, AL 35233, USA; egarner@uabmc.edu

**Keywords:** neuroblastoma, cancer stem cell, cancer-associated fibroblasts, hypoxia

## Abstract

Neuroblastoma, a solid tumor arising from neural crest cells, accounts for over 15% of all pediatric cancer deaths. The interaction of neuroblastoma cancer-initiating cells with their microenvironment likely plays an integral role in the maintenance of resistant disease and tumor relapse. In this review, we discuss the interaction between neuroblastoma cancer-initiating cells and the elements of the tumor microenvironment and how these interactions may provide novel therapeutic targets for this difficult to treat disease.

## 1. Neuroblastoma

Neuroblastoma is the most common extracranial pediatric solid tumor, accounting for nearly 15% of pediatric cancer-related deaths. Arising from embryonic neural crest cell origin, neuroblastoma may occur anywhere that sympathetic neural tissue is found, but most frequently occurs in the adrenal medulla [1]. Risk factors contributing to poor prognosis include amplification of the *MYCN* gene, age over 18 months, advanced stage and unfavorable histology [2]. Unfortunately, nearly half of patients present with metastatic disease at diagnosis [3,4], and more than 60% present with high-risk tumors that are difficult to treat [5]. The long-term survival for high-risk neuroblastoma remains at 40%, despite intensive chemotherapy, radiation and surgical therapies [6]. Management of this malignancy remains a challenge.

## 2. Tumor Microenvironment

Over the past decade, it has become increasingly evident that cancers are closely associated with a dynamic biological landscape consisting of neighboring cells, molecules and vascular and lymphatic networks. The relationship between tumor cells and noncancerous cells and proteins is referred to as the tumor microenvironment, and these components interact to each modulate the other. The effect of the microenvironment on tumor progression is varied and may prevent or promote carcinogenesis. Several studies have demonstrated that the tumor microenvironment may have anticancer properties [7,8]. In the early stages of tumor development, the microenvironment provides a physical barrier against tumorigenesis [9]. Macrophages, lymphocytes and natural killer (NK) cells play a role in this tumor suppression [10]. As the cancer progresses, the neoplastic cells may reprogram the surrounding cells and molecules in such a way to create a supportive microenvironment that promotes both tumor growth and metastasis [11]. In fact, tumor metastasis requires interaction between cancerous and noncancerous cells of the microenvironment at both the primary and secondary tumor sites [12].

## 3. Cancer Stem Cells

Another evolving paradigm in cancer biology is the concept of the cancer stem cell. Cancer stem cells (CSCs) are a small subset of cancer cells that demonstrate properties similar to normal stem cells; the capacity for self-renewal, multi-potency, proliferation and tumor maintenance [13]. The American Association for Cancer Research (AACR) has defined CSCs as cells within a tumor that “possess the capacity for self-renewal and to cause the heterogeneous lineages of cancer cells that complete the tumor” [13]. Therefore, cells must experimentally recapitulate the generation of a continuously growing tumor [13]. Many researchers have described populations of putative cancer stems cells referred to as tumor-initiating cells or tumorigenic cells. Cancer stem cells are thought to play a central role in tumor initiation, progression and recurrence [14], as well as in the development of resistance to chemotherapy [15,16] and radiation [17]. The ability to evade these interventions may arise from several mechanisms. First, this resistance occurs secondary to the ability of CSCs to regenerate, accumulate mutations and differentiate into chemoresistant cells [18]. Secondly, CSCs are capable of quiescence and may be protected from cytotoxic therapy that targets rapidly-dividing cells [19]. Several other mechanisms for chemoresistance have also been described [15,16]. Due to their resistance to chemotherapy, many believe CSCs are primarily responsible for relapse and poor survival in neuroblastoma [20]. If CSCs are the primary cells responsible for tumor growth, chemoresistance and recurrence, then therapies will need to effectively target this population of cells for a cure. Further investigation of CSCs and their interaction with the microenvironment may contribute to the development of novel therapies and improved outcomes in the management of neuroblastoma.

## 4. Neuroblastoma Heterogeneity and I-Type Cells

Neuroblastomas are heterogeneous tumors with phenotypic variants. In 1995, Ross *et al.* described a subpopulation of intermediate (I-type) neuroblastoma cells that shared characteristics with neuroblastic (N)- and substrate-adherent (S)-type cell populations. These I-type cells differentiated into either N or S cells [21]. I-type cells may represent malignant neural crest stem cells, as they demonstrate both capacity for self-renewal and multi-potency [22]. I-type cells also had a four- to five-fold higher colony forming efficiency in soft agar, a six-fold higher tumorigenicity in athymic mice and expressed the stem cell marker proteins CD133 and c-kit/CD117 [22]. These highly tumorigenic I-type cells were concluded to represent a neuroblastoma stem cell population based on their malignant and differentiation potential [23].

## 5. Further Characterization of CSCs in Neuroblastoma

CSCs were originally described in leukemia [24], but have since been identified in many different solid tumors, including neuroblastoma [20,22]. The identification of CSCs can be challenging and has not been clearly defined. Cancer stem cell populations may be distinguished from other tumor cells using several phenotypic characteristics, including the expression of certain cell-surface proteins (e.g., CD114, CD133), as well as biological properties similar to those seen in normal stem cells (Table 1) [15].

Many potential CSC surface markers have been proposed over the years. Nestin and ABCG2 are neural precursor markers and were some of the earliest markers used to describe CSCs in neuroblastoma. Adenosine triphosphate-binding cassette (ABC) transporters are transmembrane proteins involved in the efflux of a variety of chemotherapeutic drugs and may lead to chemotherapeutic resistance [25,26]. ABCG2, a member of the ABC protein family, is associated with primitive stem cells, including neural stem/progenitor cells [26]. ABCG2 is thought to play a role in maintaining stemness in these cells, as ABCG2 levels are downregulated during differentiation [26]. Nestin is a neuronal stem cell protein that has also arisen as a putative marker of CSCs [27]. In a study of human neural stem/progenitor cells, the ABCG2+ population was shown to mimic the nestin+ population in cells forming neurospheres [26]. ABCG2 and nestin staining also colocalized to the same cells [26]. Both ABCG2 and nestin have since been used as markers for putative CSCs in neuroblastoma [25,28,29].

CD133, or prominin-1, is a transmembrane glycoprotein with unclear function. CD133 was originally described as a marker for hematopoietic stem and progenitor cells [30] and has since been used as a marker of CSCs in numerous malignancies [31,32], including neuroblastoma [32,33,34], and remains the most common marker used to identify CSCs in pediatric malignancies [35]. Several investigators have sought to further evaluate the role of CD133 in neuroblastoma. Kamijo and colleagues showed CD133 expression in seven of 20 neuroblastoma cell lines studied [33]. CD133+ cells more readily formed colonies in soft-agar; CD133 knockdown led to decreased proliferation; and CD133 promoted tumor-sphere formation [33]. Cournoyer separated CD133+ and CD133− neuroblastoma cell populations using magnetic beads and then performed neurosphere and colony formation assays [34]. The CD133+ population had significantly more neurospheres and colonies on soft agar than the CD133− population. In addition, 500 CD133+ and CD133− neuroblastoma cells were injected into mice; primary tumors formed in the mice injected with CD133+ cells, but not in those injected with CD133− cells [34]. Finally, CD133 may be an independent prognostic factor for poor survival in neuroblastoma. Immunostaining for CD133 in 238 human neuroblastoma samples revealed that patients with CD133+ tumors had worse three-year event-free survival and overall survival than those with CD133− tumors [36]. These studies indicated that there was a population of stem-like cells in neuroblastoma, and this population of cells appeared to be an important factor in patient outcome.

Other studies have described side populations (SP) of cells that are sorted by fluorescence-activated cell sorting (FACS) based on their ability to exclude Hoechst 33342 dye. These SP cells express ABC transporters that are responsible for chemotherapeutic resistance [25]. Interestingly, SP appear to be enriched in stem cells [37]. A study of the SP cells in neuroblastoma demonstrated certain phenotypic features consistent with neural crest progenitor cells, including high expression of GD2 and c-kit/CD117, as well as replicative potential [25]. Hayashi and colleagues also demonstrated a small percentage of SP, or a possible stem-like population, in neuroblastomas [38]. Another study evaluated the SP of three pairs of neuroblastoma cells lines both at pretreatment and again at relapse after multimodal therapy [39]. The SP increased in the relapsed cell lines when compared to the paired pretreatment lines, and the relapsed group demonstrated greater proliferation and colony-forming ability [39]. It is believed that this population of stem-like cells is not efficiently targeted by therapy and may be a source of treatment failure and relapse in neuroblastoma.

Other populations of neuroblastoma cells that do not have CD133 expression may behave in a manner consistent with CSCs. Hansford *et al.* isolated a population of neuroblastoma cells from bone marrow metastases that were described as sphere-forming cells expressing markers of neural crest stem cells that had the capacity for self-renewal and differentiation into the cells types seen in neuroblastoma [20]. The tumor spheres from high-risk neuroblastoma samples had a greater capacity for self-renewal as they formed spheres on 1–15 serial passages (median six passages) in culture, compared to the low-risk neuroblastoma samples [20]. They also demonstrated that as few as 10 high-risk sphere-forming neuroblastoma cells could form tumors in immunodeficient mice and that these tumors could be serially passaged [20]. All of these properties were consistent with CSCs, but these cells did not express CD133, and no side population was identified.

CD114, a granulocyte colony-stimulating factor (G-CSF) receptor, is also a marker of a stem cell-like subpopulation in neuroblastoma. Hsu *et al*. described a CD114+ subpopulation in neuroblastoma that expressed genes consistent with an immature, neural crest phenotype [40]. In this study, the isolated CD114+ subpopulation was found to be 10-times more tumorigenic than the CD114− population based on limiting dilution assays, and as few as 10 CD114+ cells formed tumors in NOD/SCID mice [40]. This CD114+ cell population did not segregate into side populations based on Hoechst dye and did not co-express CD133, leading the authors to conclude that those subpopulations may lie within the more differentiated CD114− population of cells [40].

Increased aldehyde dehydrogenase (ALDH) activity has been associated with CSCs in several cancers [41], including neuroblastoma [42], where ALDH activity and expression of certain ALDH isoforms (*i.e.*, ALDH1A2, ALDH1L1, ALDH3B2) were associated with sphere and colony formation [42]. ALDH1A2 expression was also associated with increased neuroblastoma growth *in vivo*, resistance to 13-*cis*-retinoic acid and worse prognosis in neuroblastoma patients [42].

Many other cell surface markers have been used to described putative CSCs in neuroblastoma, including c-kit/CD117 [22,25,28] and Frizzled receptor 6 (FZD6) [43]. The identification of a definitive CSC population remains one of the major challenges in CSC research. Unfortunately, a specific marker or set of markers for CSCs in neuroblastoma has not been established. The ability to distinguish this tumor-initiating population from the surrounding tumor cells and normal cells will be integral to developing therapies that specifically target them. Table 1 summarizes the markers used to describe CSCs mentioned in this review.

**Table 1:** Markers used to describe putative CSCs in neuroblastoma.

	Marker	Reference
Cell surface markers	CD133	[22,28,33,34]
	CD114 (G-CSF receptor)	[40]
	CD117 (c-kit)	[22]
	ABCG2	[25,26,28]
Cytoplasmic and nuclear proteins	Nestin	[27]
	GD2	[25]
	Fzd6	[43]
Other properties/enzymes	SP	[25,29,38,39]
	ALDH	[41,42]

## 6. The Microenvironment and the CSC Niche

The tumor microenvironment is an important regulator of stem cell differentiation and tumorigenesis. In order to discuss the involvement of CSCs in tumor progression, it is important to understand the special microenvironment, or CSC niche, necessary for these cells to establish and maintain their stemness [44,45]. This niche has been characterized by hypoxia [46], pro-inflammatory signals [47], acidic stress [48] and extracellular matrix remodeling [49]. All of these factors work in concert to promote CSC self-renewal and prevent differentiation. Although many of these mechanisms are still being explored, some of the known interactions of CSCs with the neuroblastoma microenvironment will be discussed in the following sections.

## 7. Hypoxia and Neuroblastoma CSCs

Stem cell research has demonstrated that hypoxia is part of the specialized niche for CSCs. Hypoxia promotes a stress response that shifts cells toward pro-survival pathways that are regulated by hypoxia inducible factor-α (HIFα) proteins [50]. Through these pathways, hypoxia creates a microenvironment favoring poorly-differentiated tumor cells [51]. In a study examining several solid tumors, including neuroblastoma, Yeger and colleagues demonstrated that *in vitro* exposure to hypoxic conditions significantly increased the SP fraction [52]. Furthermore, by creating an injured conditioned medium model derived from hypoxic bone marrow stromal cells, they demonstrated that SP cells migrated toward the hypoxic zones, suggesting hypoxia served as an important contributor to the CSC niche [52]. Hypoxia caused de-differentiation of human neuroblastoma cells toward an immature and neural crest-like phenotype [52]. In another study, neuroblastoma cells exposed to hypoxia had an upregulation of hypoxia-induced genes, as well as an upregulation of neural crest marker genes, including c-kit and Notch-1 [53]. These phenotypic changes were hypothesized to be secondary to upregulation of hypoxia-inducible factor (HIF) 1α and HIF-2α. HIF-2α was later correlated with advanced clinical stage and worse prognosis in neuroblastoma [54]. HIF-2α has also been associated with regulation of several stem cell-associated genes [55,56,57]. Pietras *et al.* demonstrated that the knockdown of HIF-2α decreased VEGF expression and led to partial sympathetic neural differentiation of neuroblastoma stem cells [55]. These studies suggested that hypoxia and HIF pathways contributed to neuroblastoma progression in part by the maintenance of immature, stem-like tumor cells.

Another possible location for CSCs is in a perivascular niche originally described in brain tumor stem cells [58]. This niche was discovered when Nestin+/CD133+ cells were found along the capillaries of brain tumors. Cocultures of the Nestin+/CD133+ cells with primary human endothelial cells were shown to maintain a greater population of self-renewing, undifferentiated brain tumor cells compared to the control group [58]. These findings led the investigators to conclude that secreted factors from the endothelial cells were responsible for maintaining the stem-like state. A perivascular CSC niche has also been described in neuroblastoma [59]. A small subset of HIF-2α+ neuroblastoma cells were found in the perivascular space. These cells lacked sympathetic differentiation markers, but expressed immature neural crest markers, including Notch-1, HES-1 and c-kit [59]. This information suggests that targeting the perivascular niche with anti-angiogenic therapies may disrupt this microenvironment and lead to loss of stemness in these cells.

## 8. Cancer-Associated Fibroblasts and the Extracellular Matrix

Cancer-associated fibroblasts (CAFs) play an important role in promoting tumor growth, invasion and angiogenesis [10,60]. CAFs are activated fibroblasts that produce extracellular matrix (ECM), proteinases, cytokines, chemokines and growth factors [61]. CAFs may arise from local fibroblasts or other progenitor cells, including bone marrow-derived cells, endothelial cells and epithelial cells via mesenchymal transition [10]. Unlike normal fibroblasts, CAFs remain in an activated state and do no return to a normal phenotype or undergo apoptosis [62]. In 2009, Zeine and colleagues evaluated CAFs in 60 primary neuroblastoma tumors [60]. CAFs were identified by positive immunostaining for the CAF-marker α-SMA, and a >1.0% area of positive staining was considered high. Increased CAFs were associated with significantly higher microvascular proliferation and Schwannian stroma-poor histology, both poor prognostic factors [60].

Matrix metalloproteinases (MMPs) are zinc-containing endopeptidases that remodel the ECM and have been implicated in tumor growth, invasion and metastasis [63]. MMPs are often overexpressed by CAFs [62]. In addition to ECM degradation, cleaved products of MMPs, such as fibronectin and collagen, serve as chemotactic factors for inflammatory cells [64] and play a role in angiogenesis [65]. A role for matrix metalloproteinase-9 (MMP-9) has been described in neuroblastoma. When neuroblastoma tumor cells were implanted into MMP-9-deficient mice, the tumor vasculature appeared to be inhibited [65]. DeClereck and colleagues demonstrated that MMP-9 was involved in the recruitment of bone marrow-derived leukocytes into the tumor microenvironment [63]. This group also discovered that neuroblastomas with unfavorable histology and advanced disease have more inflammatory cells expressing MMP-9 [63,66]. MMP-14 overexpression has also been correlated with aggressiveness and poor outcome in neuroblastoma [67], and MMP-14 knockdown decreased migration, invasion and angiogenesis in neuroblastoma cells [67]. The role of MMPs in ECM remodeling is still being explored.

## 9. Inflammatory and Immune Cells of the Microenvironment

Inflammatory and immune cells are active participants in the tumor microenvironment [68], either promoting or suppressing tumorigenesis. Macrophages, lymphocytes and NK cells are involved in this balance [68,69,70,71]. After transformation, the immune cells are often redirected by tumor cells to take on a pro-angiogenic and immunosuppressive state [72]. Recent data suggest that the interaction between neoplastic cells and inflammatory cells may contribute to a metastatic phenotype in neuroblastoma [69].

Macrophages are seen within most solid tumors, and high tumor-associated macrophage (TAM) content is associated with worse prognosis [69,72,73]. In an immunohistochemistry analysis of 71 neuroblastoma tumors using the macrophage marker CD163, Seeger and colleagues demonstrated a greater concentration of TAMs in metastatic neuroblastomas when compared to locoregional disease [69]. The same group performed gene expression studies in *MYCN* nonamplified neuroblastomas, and identified a 14-gene signature consisting of both inflammatory and tumor cell genes that could predict disease progression. This model consisted of five TAM-related genes, including CD14, CD33, FCGR3 (CD16), interleukin-6 receptor (IL6R) and interleukin-10 (IL10), which contributed to about 25% of the accuracy of this 14-gene scoring model [69]. A subsequent study confirmed that higher levels of TAM-specific genes (CD14, CD16, IL6, IL6R and TGFB1) were associated with a worse prognosis in MYCN-nonamplified neuroblastomas [73].

Natural killer (NK) cells and natural killer T (NKT) cells are cytotoxic lymphocytes of the innate immune system present in the tumor microenvironment. NK cells are potent anti-tumor cells [74] and have displayed strong cytotoxic activity against neuroblastoma, both *in vitro* [75] and *in vivo* [76]. Castriconi and others evaluated the cytotoxic effects of NK cells on freshly-isolated neuroblastoma cells and discovered that susceptibility to NK-mediated lysis correlated with the poliovirus receptor (PVR/CD155) [77]. Invariant natural killer T (iNKT) cells also play a role in antitumor immunity. These cells migrate toward neuroblastoma tumor cells in a CCL2-dependent manner, and CCL2 expression is inversely associated with *MCYN* amplification [78]. The survival curve for patients with iNKT^+^ *versus* iNKT^−^ tumors was similar to the survival curve for *MCYN* non-amplified and *MYCN* amplified tumors [78]. Given the ability of these immune cells to attack human tumor cells, significant attention has been paid to NK-cell-based immunotherapies for cancer treatment [78,79,80].

In neuroblastoma, an immunosuppressive microenvironment may lead to the suppression of the anti-tumor capabilities of immune cells [71,81]. IL-6, secreted by monocytes, and TGFβ1, secreted by neuroblastoma cells and monocytes, were shown to suppress IL-2 activation of NK cells [81]. Myeloid-derived suppressor cells (MDSCs) comprise another population of tumor-infiltrating immune cells that promote tumor growth. These immature myeloid cells are attracted to the tumor by soluble factors released in the microenvironment and are responsible for immunosuppressive and tumor-promoting activity [70,82]. Santilli and colleagues first described this population of cells in neuroblastoma [82]. They demonstrated that inhibition of MDSCs in immunocompetent, but not immunodeficient mouse models of neuroblastoma, resulted in inhibition of tumor growth [82].

## 10. Cytokines, Chemokines and Other Signaling Pathways

Cytokines and other inflammatory mediators in the microenvironment influence tumor progression. Chemokines contribute to the recruitment and function of specific types of lymphoid and myeloid cells [78]. In addition, some believe the same homing and mobilization mechanisms used by normal stem cells may also be involved in the process of cancer stem cell metastasis [83]. CXCR4 is a chemokine receptor highly expressed on tumor cells, and activated CXCR4 can directly stimulate cancer cell proliferation [62,84]. This receptor and its ligand, CXCL12, also known as stromal cell-derived factor-1 (SDF-1), appear to play a role in tumor metastasis. The expression of CXCR4 by CSCs may lead to the metastasis to organs that have a high expression of the ligand SDF-1, including bone, lung, lymph nodes and liver [83]. This receptor and its ligand have been found to promote neuroblastoma cell migration and bone marrow metastasis in neuroblastoma [85]. Russell *et al.* demonstrated that higher CXCR4 expression was associated with advanced stage neuroblastoma tumors and in patients with cortical bone and bone marrow metastasis [86]. CXCR4-overexpressing cells were also shown to be associated with increased incidence of bone marrow metastases in an *in vivo* neuroblastoma xenograft model [87].

Other signaling pathways appear to be involved in the CSC niche, but their role has not been clearly defined. Notch, Wnt and Sonic Hedgehog (SHH) are developmental signaling pathways involved in embryonic and postembryonic stem cell self-renewal that have also been implicated in tumorigenesis. The Notch pathway is involved in neural development [88], may regulate cancer stem cells [89] and is involved in neuroblastoma cell proliferation [90]. The Sonic Hedgehog (SHH) signaling pathway influences neural crest cell development, and activation of this pathway affects neuroblastoma proliferation [91]. The Wnt pathway is implicated in chemoresistance in CD133+ neuroblastoma cells [92]. All of these pathways and others will need to be studied more extensively.

## 11. CSC Targeted Therapy

**Figure 1:**
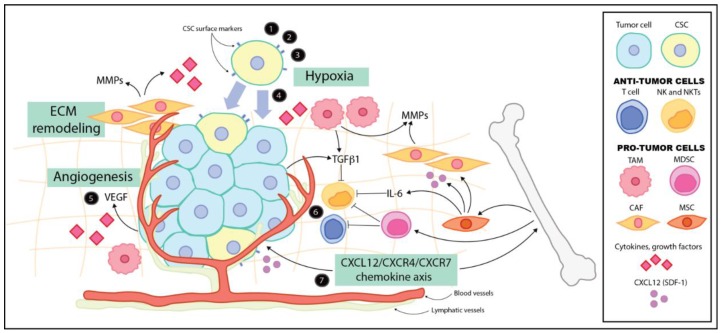
Neuroblastoma tumor microenvironment and potential sites of therapy. (1) Embryonal signaling pathways (e.g., Notch, Hedgehog, Wnt); (2) cancer stem cell (CSC) surface marker-directed therapy (e.g., CD133, CD114, nestin, *etc.*); (3) selective inhibition of CSCs (e.g., DECA-14, rapamycin); (4) differentiation therapy (e.g., retinoic acid, proteasome inhibitors); (5) blocking angiogenesis/VEGF/VEGFR (e.g., Bevacizumab); (6) immunotherapy and immune activation (e.g., lenalidomide, ch14.18, GM-CSF, IL-2); (7) blocking chemokine/receptor function (e.g., CXCL12/CXCR4/CXCR7 chemokine axis). TAM, tumor-associated macrophage; MDSC, myeloid-derived suppressor cell; CAF, cancer-associated fibroblast.

CSCs may be responsible for chemoresistance and tumor relapse, leading researchers to develop therapies directed toward this population. Several challenges exist in targeting CSCs. First, identification of this population may be difficult, as cell surface markers, such as CD133, may not be present on all CSCs [20]. Secondly, the potential CSC targets, such as surface markers, signaling pathways and the microenvironment, are also relevant to normal stem cells and may not represent a specific target for CSCs [30,35]. Several potential therapies that target the CSC population while sparing the normal stem cell population are being explored in neuroblastoma. Kaplan and colleagues employed small molecule screening assays to identify two agents, DECA-14 and rapamycin, that selectively targeted neuroblastoma stem-like cells while avoiding the normal stem cell population [93]. Mahller *et al.* used Nestin-targeted oncolytic herpes simplex virus (oHSV) and were able to kill both differentiated and tumor initiating neuroblastoma cells [29]. Another potential mechanism of therapy is using differentiating agents, such as retinoids. One group treated neuroblastoma cells with 13-*cis*-retinoic acid and the proteasome inhibitor MG132 [94]. These agents alone or in combination lead to decreased expression of stem cell markers (*i.e.*, Nestin, Sox2, Oct4) and inhibited sphere formation [94]. Alternatively, the tumor microenvironment may be an effective target in neuroblastoma with therapies directed at cytokines, growth factors and immune cells. Angiogenesis may be targeted with the anti-VEGF antibody Bevacizumab [95]. Several immunotherapies are also being explored [5,71,81,96] (Figure 1). Other proposed future therapies are the embryonic signaling pathways, including Notch, EGFR, Wnt and SHH [35,91].

## 12. Conclusions

CSCs likely play an important role in the progression and recurrence of neuroblastoma. Identification of this small subset of cells remains challenging. Further investigation of CSCs and the specialized microenvironment in which they thrive may provide novel therapeutic targets for neuroblastoma.
